# Supplementary material for: Estimation of country-level incidence of early-onset invasive Group B Streptococcus disease in infants using Bayesian methods
Source: PLoS Comput Biol. 2021 Jun 14;17(6):e1009001. doi: 10.1371/journal.pcbi.1009001 (PMC8202927; doi:10.1371/journal.pcbi.1009001)

*S1 Appendix*

**Estimation of country-level incidence of early-onset invasive Group B Streptococcus disease in infants using Bayesian methods**

**Authors:** Bronner P. Gonçalves^1,2,*^, Simon R. Procter^1,2^, Sam Clifford^1^, Artemis Koukounari^1,2^, Proma Paul^1,2^, Alexandra Lewin^3^, Mark Jit^1#^, Joy Lawn^1,2#^

**Affiliations**

^1^ Department of Infectious Disease Epidemiology, London School of Hygiene & Tropical Medicine, London, UK

^2^ Maternal, Adolescent, Reproductive & Child Health (MARCH) Centre, London School of Hygiene & Tropical Medicine, London, UK

^3^ Department of Medical Statistics, London School of Hygiene & Tropical Medicine, London, UK

^#^ Equal contribution

* Corresponding author

**Table of contents**

1. *Prior predictive distributions*
2. *Sensitivity analyses*
3. *Supplementary Table (Table A)*
4. *Supplementary Figures (Figures A – J)*

**Prior predictive distributions**

We sampled from prior predictive distributions using the prior assumptions described in the *Results* section (see **Fig B** and **C**). For the maternal GBS colonization model, the following priors were used:

$$\mu_{g} \sim Normal (-1, 1)$$

$$\beta_{c} \sim Normal (0, 1)$$

$$\beta_{s} \sim Normal \left( 0, 1 \right)$$

$$\sigma_{c} \sim Uniform (0, 5)$$

$$\sigma_{s} \sim Uniform (0, 5)$$

The matrices with study-level and country-level predictors, including all covariates, as well as the study sample sizes were used.

For the model on the risk of early-onset invasive GBS disease in babies born to colonized mothers, we used the following priors:

$$\alpha_{g} \sim Normal (-4, 1)$$

$$\beta_{IAP} \sim Normal (0, 1)$$

$$\sigma_{eo} \sim Uniform (0, 5)$$

We generated data with the same number of studies as in the dataset used in the analysis. Study-specific antibiotic coverage was used.

**Sensitivity analyses**

In addition to the analyses with the priors reported in the *Results* section, we performed sensitivity analyses to assess the effect of different prior assumptions. In the table below, we show posterior estimates for key parameters assuming different prior distributions. We performed sensitivity analyses for regression coefficients, standard deviations as well as reporting parameter, changing one prior each time, as opposed to one model with all the changes

|  |  |  |  |  |
| --- | --- | --- | --- | --- |
| **Model component** | **Parameter** | **Sensitivity Analysis** | **Posterior median (95% interval) – Main analysis** | **Posterior median (95% interval) – Sensitivity analysis** |
|  |  |  |  |  |
| *Maternal GBS colonization* |  |  |  |  |
|  | *µ_g_* | Normal (0, 1) | -1.66 (-1.78, -1.53) | -1.65 (-1.78, -1.53) |
|  | *σ_c_* | Cauchy (0, 5) | 0.33 (0.23, 0.45) | 0.33 (0.23, 0.45) |
|  | *σ_s_* | Cauchy (0, 5) | 0.52 (0.47, 0.58) | 0.52 (0.47, 0.58) |
|  | β coefficients | Normal (0,10) | See Table 1 | Only minor changes in coefficients |
|  |  |  |  |  |
| *EOGBS risk in colonized mothers* |  |  |  |  |
|  | *α_g_* | Normal (-4, 5) | -4.12 (-4.92, -3.42) | -4.14 (-5.01, -3.36) |
|  | *σ_eo_* | Cauchy (0, 5) | 1.02 (0.59, 1.72) | 1.04 (0.59, 1.73) |
|  |  |  |  |  |
| *Incidence* |  |  |  |  |
|  | γ (reporting) | Beta (1, 1) | 0.59 (0.34, 0.89) | 0.63 (0.34, 0.96) |
|  |  |  |  |  |

We also fit the model on early-onset invasive GBS disease risk using all studies reported in the review by Russell et al (see *Methods* section), including two studies that excluded preterms. In this secondary analysis, the posterior medians (95% intervals) of *α_g_* and *β_IAP_* are -4.25 (-5.00, -3.50) and -0.03 (-0.05, -0.02), respectively.

**Supplementary Table**

**Table A.** Maternal GBS colonization regression coefficients at the study and country levels and standard deviation parameters. Results of three models are presented: estimation based on the maternal GBS colonization hierarchical model (Independent model , as in **Table 1** of the main text); estimation based on the full model that also incorporates data on risk and incidence data from selected studies (full model I); estimation that incorporate data from a larger number of incidence studies (full model II, see *Synthesis with incidence data* section).

|  | **Independent model** | | **Full model I** | | **Full model II** | |
| --- | --- | --- | --- | --- | --- | --- |
|  | Median | 95% interval | Median | 95% interval | Median | 95% interval |
| **Country-level covariates** |  |  |  |  |  |  |
| *Percent coverage of ATB for LRI* | 0.30 | (0.07 - 0.53) | *0.30* | (0.07 - 0.54) | *0.31* | (0.07 - 0.54) |
| *Maternal education* | -0.18 | (-0.48 - 0.11) | *-0.18* | (-0.48 - 0.12) | *-0.19* | (-0.50 - 0.12) |
| *GNI* | 0.16 | (0.03 - 0.29) | *0.16* | (0.03 - 0.29) | *0.16* | (0.03 - 0.29) |
| *Neonatal mortality* | 0.22 | (0.00 - 0.44) | *0.22* | (0.01 - 0.44) | *0.23* | (0.00 - 0.45) |
| *HIV prevalence* | 0.19 | (0.06 - 0.32) | *0.18* | (0.065 - 0.31) | *0.19* | (0.05 - 0.32) |
| *Obesity prevalence* | 0.20 | (0.08 - 0.31) | 0.20 | (0.09 - 0.31) | 0.21 | (0.09 - 0.32) |
| **Study-level covariates** |  |  |  |  |  |  |
| *Swab site* | -0.23 | (-0.39 - -0.07) | *-0.23* | (-0.38 - -0.07) | *-0.23* | (-0.38 - -0.08) |
| *Selective Agar* | -0.30 | (-0.47 - -0.13) | -0.30 | (-0.47 - -0.13) | -0.30 | (-0.47 - -0.13) |
| **Standard deviation parameters** |  |  |  |  |  |  |
| *σ_c_* | 0.33 | (0.23 - 0.45) | *0.33* | (0.23 - 0.45) | *0.33* | (0.24 - 0.46) |
| *σ_s_* | 0.52 | (0.47 - 0.58) | 0.52 | (0.47 - 0.58) | 0.52 | (0.47 - 0.57) |

**Supplementary Figures**

**Figure A.** Study-specific maternal GBS colonization prevalence (i.e. percentage of study population colonized by GBS bacteria) by diagnostic combinations**.** RV = recto-vaginal sampling; SA = selective agar.


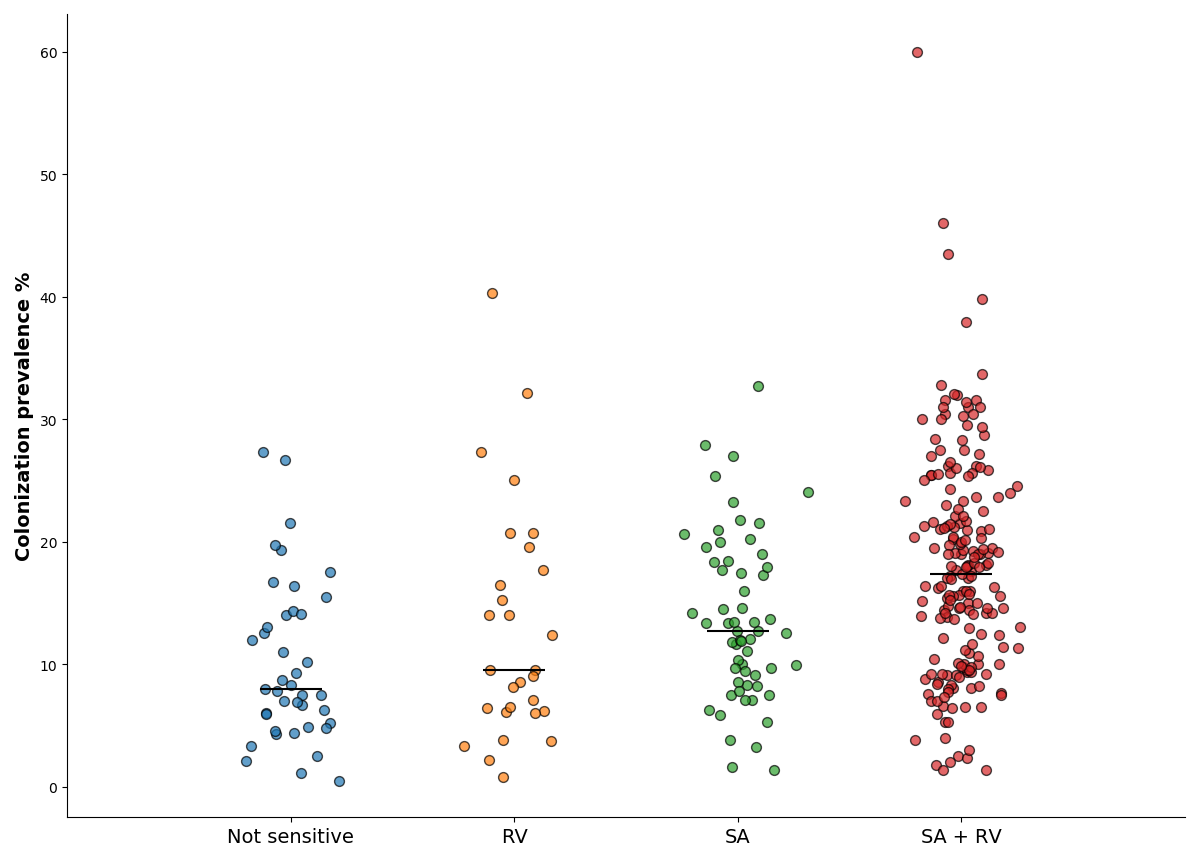


**Figure B.** Maternal GBS colonization prevalence model and prior predictive distribution. In the upper panel, medians and interquartile ranges for country-level prevalences (y-axis) are shown for 82 countries. In the bottom panel, medians and intervals for percentages of study populations colonized by GBS bacteria are shown (325 studies). Study- and country-level predictors were used in these simulations.


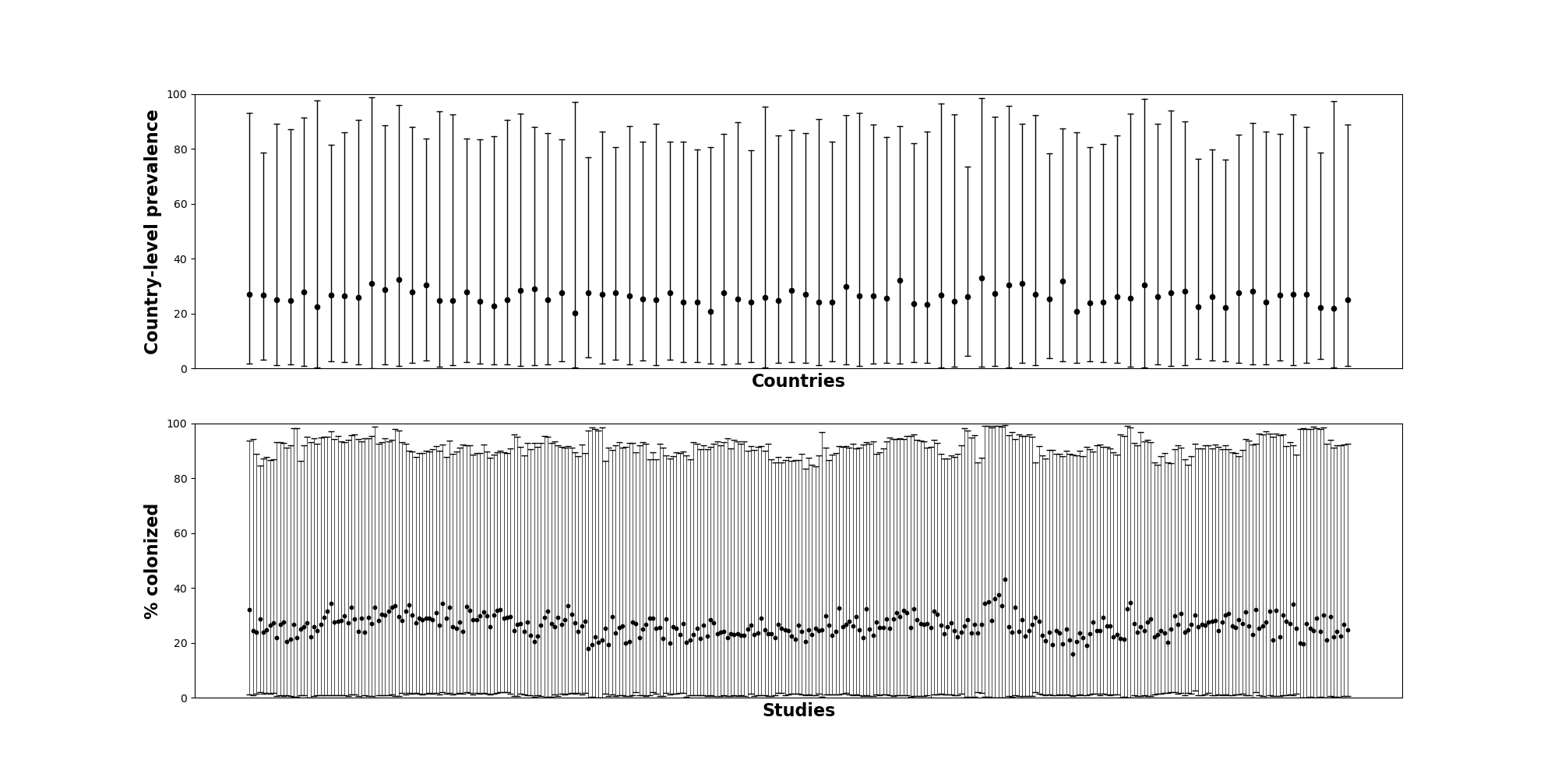


**Figure C.** Prior predictive distribution of the model on early-onset invasive GBS disease in babies born to GBS-colonized mothers. The histogram shows the distribution of the proportion of children developing early-onset invasive GBS disease (x-axis) in a study with sample size of 825, which corresponds to one of the study sizes in our analysis.


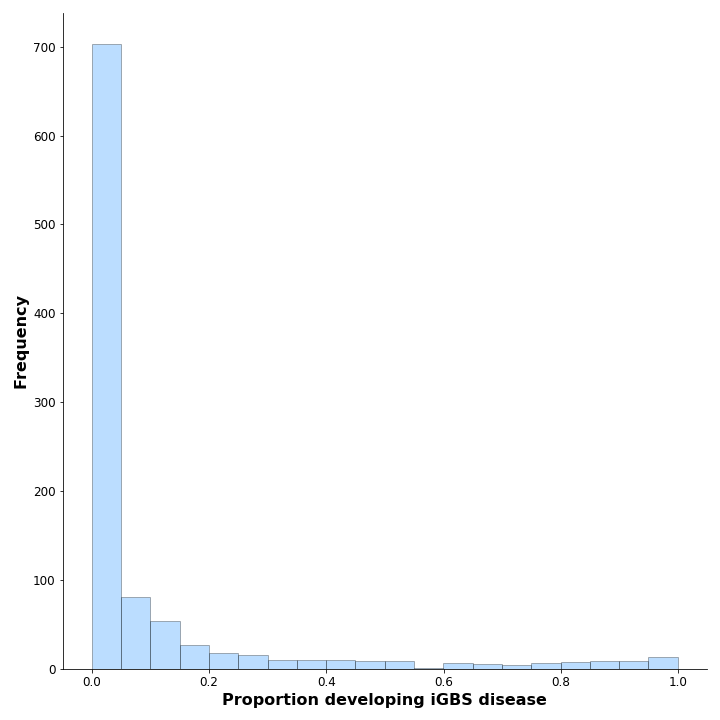


**Figure D.** Mixed predictive checks of the maternal GBS colonization prevalence model. We sampled new country-specific logit-prevalences (*μ_rep_j_*) using the values of *μ^(m)^_g_*, *β^(m)^_c_* and *σ^(m)^_c_*, where *m* corresponds to the sampler iteration *m* and the suffix ‘*_rep*’ indicates that these were used to generate replicated datasets. We then sampled$Normal \left( {\mu\_rep}_{j} +\beta_{s} X_{ij}^{S} , \sigma_{s}^{2} \right)$, using *σ^(m)^_s_*. In the upper panel, the y-axis represents the distribution of *y_rep*, the predicted number of GBS colonized mothers in each study, divided by the corresponding sizes of the 325 studies (x-axis) included in this estimation: medians are shown as horizontal lines; darker tones of each color represent interquartile ranges, and lighter shades, 95% intervals. Black circles represent observed percentages of GBS-colonized participants. Bars with the same color correspond to studies performed in the same country. Of note, the six studies with prevalence above 35% had sample size below or equal to 300 participants (mean sample size of these studies, 175 participants). In the bottom left panel, the distribution of the highest study prevalences (*y_rep* divided by study size) in replicated datasets is shown; the vertical dashed line represents the highest prevalence in the observed data. The bottom right panel presents the mean prevalence over all studies in each replicated dataset, and the dashed line corresponds to the mean prevalence in the analysis dataset.

**
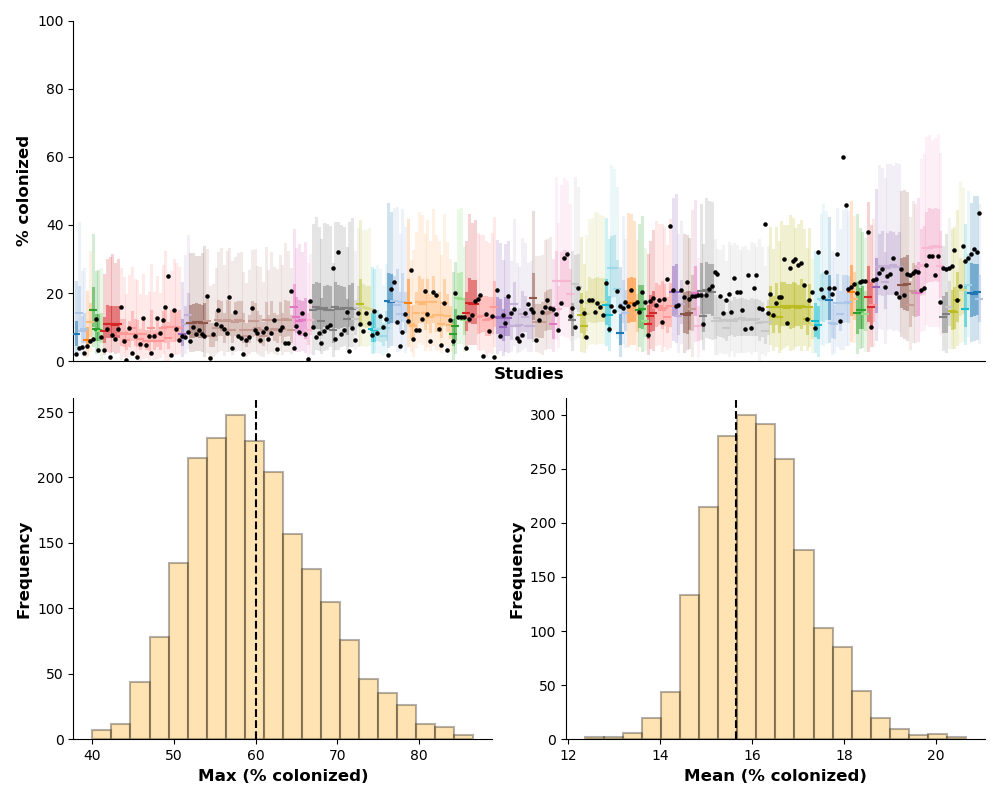
**

**Figure E.** Mixed predictive checks of the model on early-onset invasive GBS disease risk. The upper panel shows percentages of participants in each study developing invasive GBS disease (black circles), with the corresponding distribution, i.e. the predicted number of cases divided by the study size. For these calculations, data on intrapartum antibiotic use in the different studies were used. The bottom left and right panels present the distributions of maximum and mean risks in studies in replicated datasets, respectively. The vertical dashed lines correspond to the same metrics in the observed data.

**
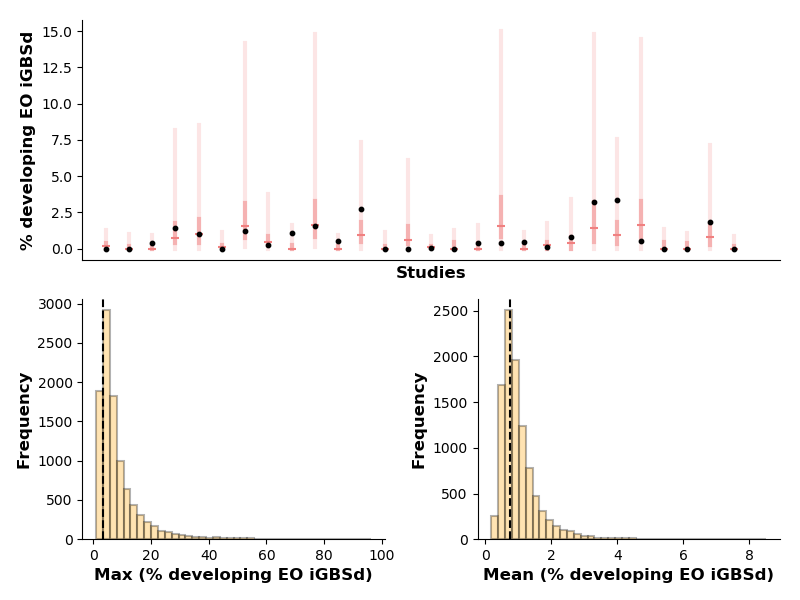
**

**Figure F.** Posterior median maternal GBS colonization prevalence (circles) estimated by the model that only used data from GBS colonization studies (x-axis) and by the full model (y-axis), that combined these data with early-onset invasive GBS disease incidence and risk data. Blue circles represent the output of the model that only included a selected set of incidence studies, and red circles, the output of the full model that included a higher number of incidence studies. Posterior interquartile ranges are also presented.


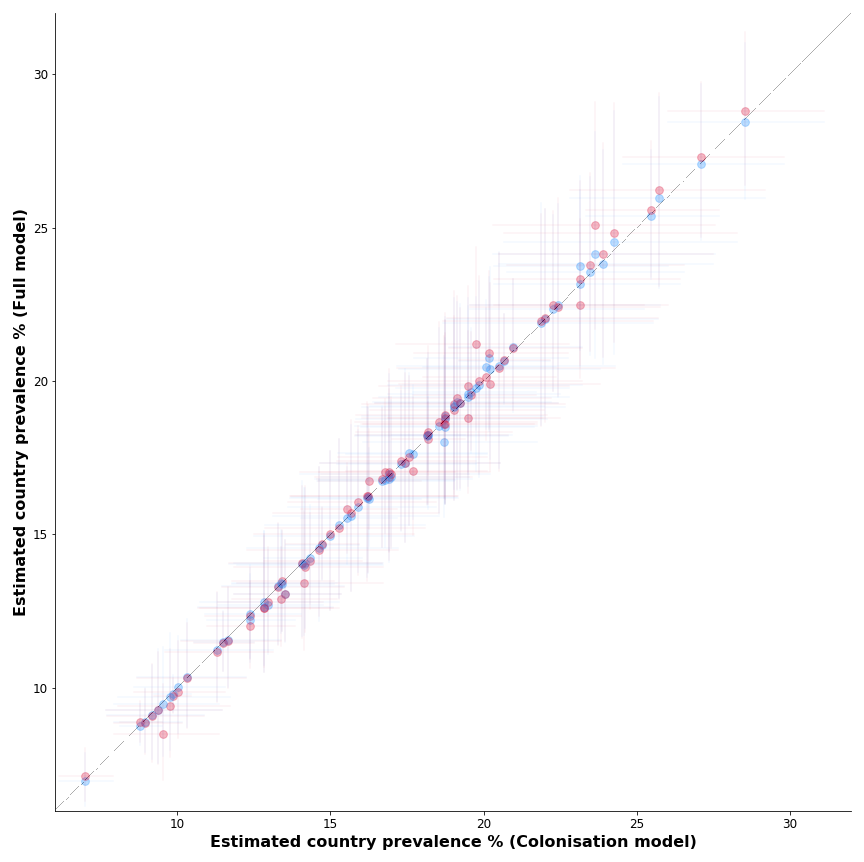


**Figure G.** Predictive checks for the full model. The upper and middle panels present graphs similar to those presented in **Figures D** and **E**. The bottom panel shows the predictive distribution for the ten incidence studies included in the analysis. Note the y-axis of the bottom panel represents the number of early-onset invasive GBS disease per 1,000 births.

**
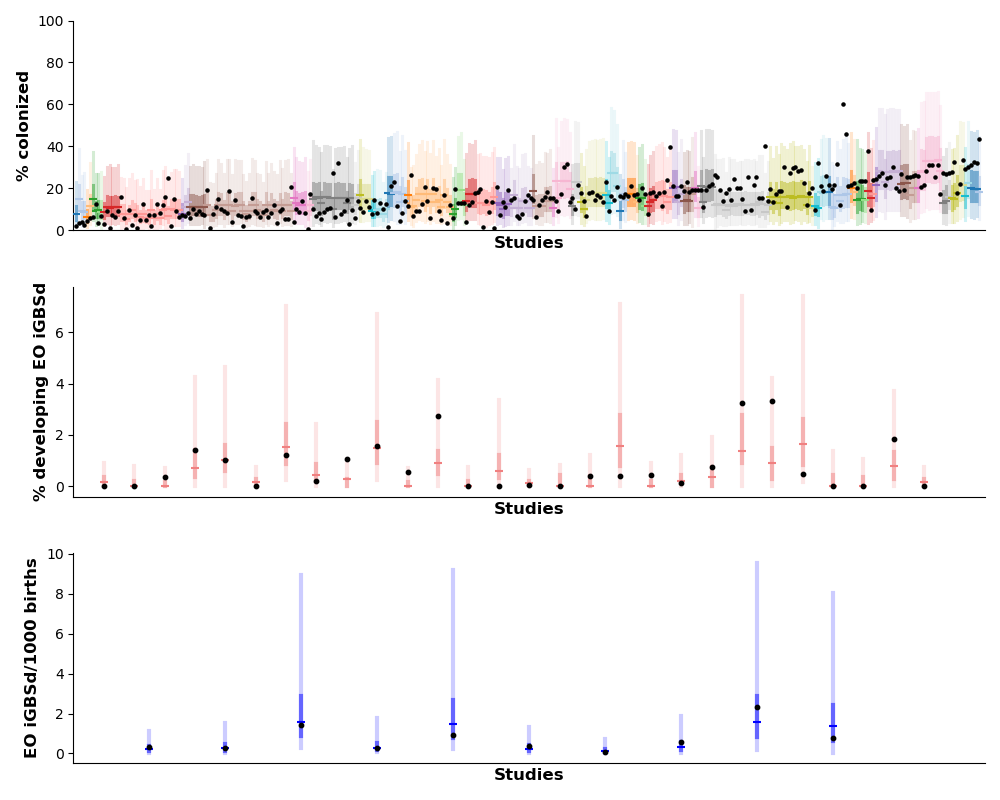
**

**Figure H.** Distribution of divergences in the centered model. Each red circle represents divergent transitions. The y-axis corresponds to the log of the values of the parameter *σ_c_*, and the x-axis, the values of the parameter *μ_j_* for one randomly selected country. When generating the data used in this graph, 2.2% of iterations diverged.


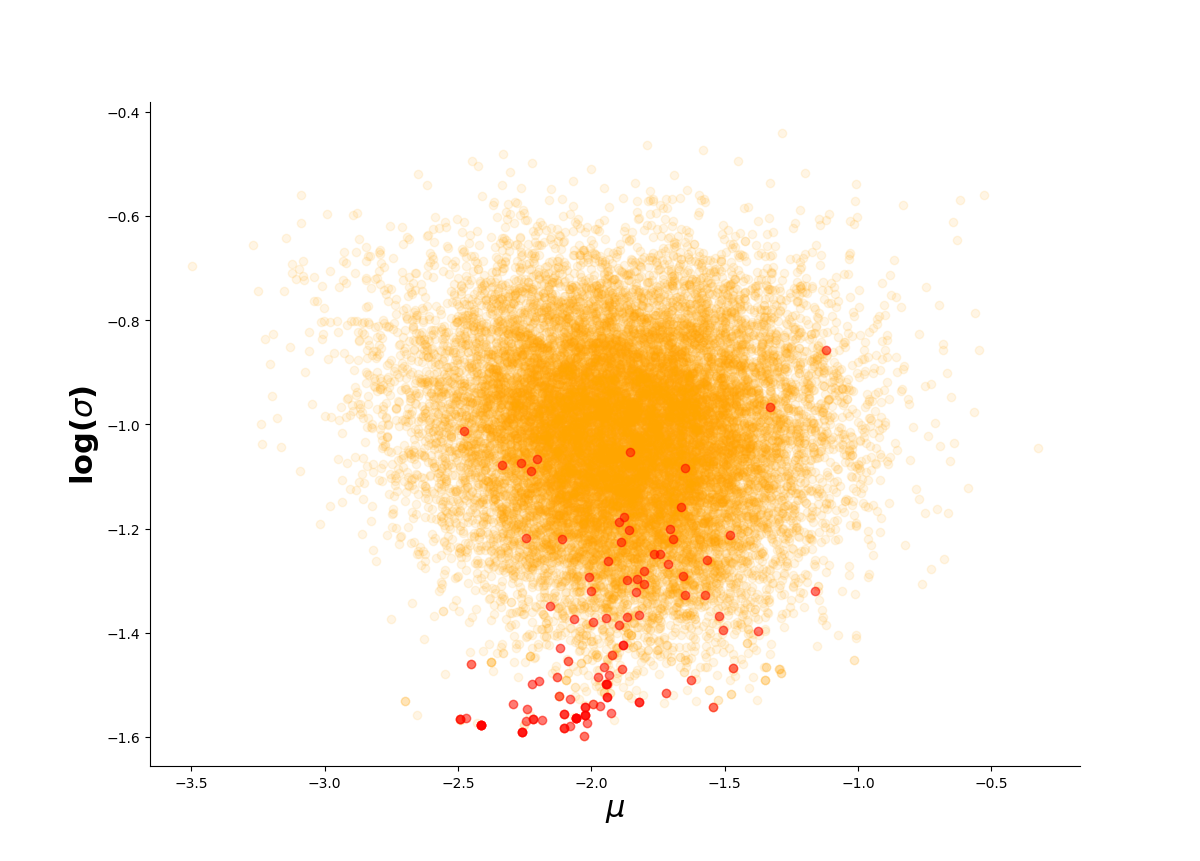


**Figure I.** Posterior distributions of regression coefficients in the hierarchical model for maternal GBS colonization prevalence that includes all variables.


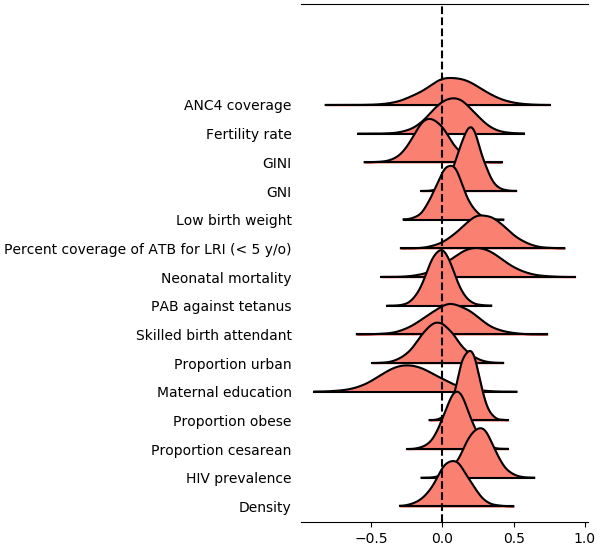


**Figure J**. Estimated country-level incidence of early-onset invasive GBS disease per 1,000 births (y-axis). Estimates for the 82 countries (x-axis) with maternal GBS colonization data are shown. Black circles represent the incidence of invasive GBS disease cases estimated in the evidence synthesis work by Seale and colleagues. Estimates from different models described here correspond to different colours: blue represents Bayesian estimates that do not use incidence data; red represents full model I; and orange, full model II. For comparability, we used the same numbers of live births per country as Seale and colleagues.


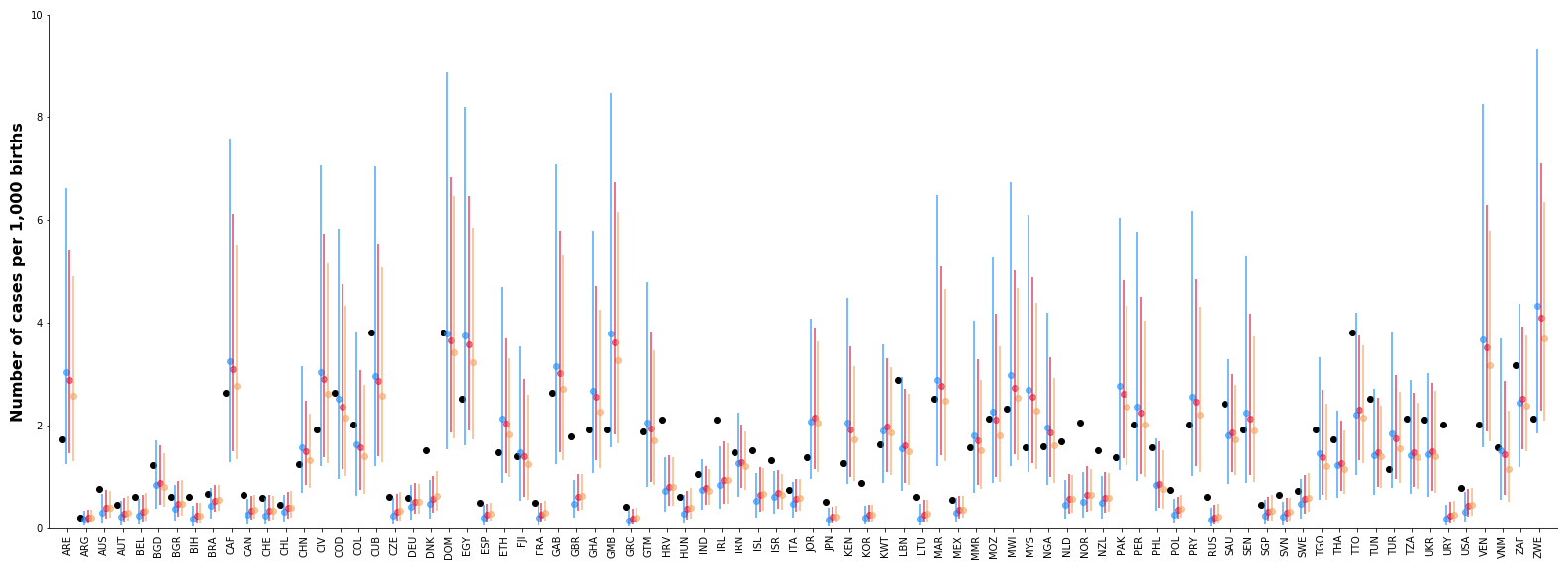

Supplement: S1 Appendix — The following tables and figures were included: Table A. Maternal GBS colonization regression coefficients at the study and country levels and standard deviation parameters. Figure A. Study-specific maternal GBS colonization prevalence (i.e. percentage of study population colonized by GBS bacteria) by diagnostic combinations. Figure B. Maternal GBS colonization prevalence model and prior predictive distribution. Figure C. Prior predictive distribution of the model on early-onset invasive GBS disease in babies born to GBS-colonized mothers. Figure D. Mixed predictive checks of the maternal GBS colonization prevalence model. Figure E. Mixed predictive checks of the model on early-onset invasive GBS disease risk. Figure F. Posterior median maternal GBS colonization prevalence estimated by the model that only used data from GBS colonization studies and by the full model, that combined these data with early-onset iGBS disease incidence and risk data. Figure G. Predictive checks for the full model. Figure H. Distribution of divergences in the centered model. Figure I. Posterior distributions of regression coefficients in the hierarchical model for maternal GBS colonization that includes all variables. Figure J. Estimated country-level incidence of early-onset invasive GBS disease per 1,000 births. (DOCX) [file pcbi.1009001.s001.docx]
