# Supplementary material for: Estimation of country-level incidence of early-onset invasive Group B Streptococcus disease in infants using Bayesian methods
Source: PLoS Comput Biol. 2021 Jun 14;17(6):e1009001. doi: 10.1371/journal.pcbi.1009001 (PMC8202927; doi:10.1371/journal.pcbi.1009001)
Supplement: Attachment — Submitted filename: Goncalves_Review_2021.pdf [file pcbi.1009001.s002.pdf]

Goncalves, et al seek to employ a Bayesian method for estimating the prevalence of invasive neonatal GBS disease. The authors combine various forms of epidemiological data to achieve this goal, including maternal colonization data.

Given the dearth of available data regarding the incidence of invasive GBS disease in neonates globally and with regards to specific populations, I find that this study addresses a critical need in the field. However, both major and minor considerations must be addressed before the manuscript is suitable for publication in *PLoS Computational Biology*. Overall, the manuscript text should be carefully edited to ensure clarity of language and style. There are several areas of the manuscript where meaning is lost due to poor grammar and confusing sentence structure.

Please find my specific comments delineated in a section-by-section fashion below.

Major Comments:

Abstract:

-Line 26-28: Will development of GBS vaccines be bolstered by this study? Please be more specific as to how this study will contribute to the field of GBS epidemiology.

Background:

-The authors should make a better case for inferring the incidence of invasive GBS disease based on alternative streams of information. Invasive GBS disease is a life-threatening illness; thus, one would expect that conclusive clinical information would readily available and highly representative of the true incidence of this disease. Please address this (either in the Abstract or further in the Background section).

-Also, the authors should expand on the need for inferred incidence of invasive GBS disease (i.e. what can this information be used for).

Results:

-Figure 5: It is unclear to me whether the data are represented as absolute counts or relative cases/population. If the former, I recommend changing the graph to report cases per population.

Discussion:

-How does this study address issues of population-specific differences within a country? Would this study be able to estimate incidence of invasive GBS disease in regions where specific ethnic and cultural minorities dominate?

-Overall, the discussion appropriately contextualizes the findings of this manuscript.

Minor Comments

Line 38-39: The parallel structure of this sentence seems incorrect or at least awkward. Please reword this sentence.

Line 372-375: Parallel structure of this sentence is lacking.

Line 412-415: Please shorten this sentence or split it in two.
