# Supplementary material for: Estimation of country-level incidence of early-onset invasive Group B Streptococcus disease in infants using Bayesian methods
Source: PLoS Comput Biol. 2021 Jun 14;17(6):e1009001. doi: 10.1371/journal.pcbi.1009001 (PMC8202927; doi:10.1371/journal.pcbi.1009001)
Supplement: Attachment — Submitted filename: Answers_to_comments_09032021.docx [file pcbi.1009001.s003.docx]

**Manuscript:** Estimation of country-level incidence of early-onset invasive Group B Streptococcus disease in infants using Bayesian methods

*Overall answer to reviewers’ comments*

We would like to thank the reviewers for their comments, that helped clarifying the motivation of this work and improved the presentation of results. Importantly, the reviewers agree that estimation of invasive GBS disease incidence is an important epidemiological question, and improved methods are necessary. We believe our paper, with the changes made to address the comments below, will contribute to an understanding of invasive GBS disease as an important public health problem globally and further development of better preventative strategies.

**Reviewer #1**

*Comment*

**Goncalves, et al seek to employ a Bayesian method for estimating the prevalence of invasive neonatal GBS disease. The authors combine various forms of epidemiological data to achieve this goal, including maternal colonization data.**

**Given the dearth of available data regarding the incidence of invasive GBS disease in neonates globally and with regards to specific populations, I find that this study addresses a critical need in the field. However, both major and minor considerations must be addressed before the manuscript is suitable for publication in PLoS Computational Biology. Overall, the manuscript text should be carefully edited to ensure clarity of language and style. There are several areas of the manuscript where meaning is lost due to poor grammar and confusing sentence structure.**

*Answer to comment*

We agree with the reviewer that there is a critical need to estimate incidence of invasive GBS disease globally, as this estimation will help to set public health priorities and quantify the potential benefits of preventative strategies such as maternal vaccination and increased coverage of intrapartum prophylaxis. We are indebted to the reviewer for carefully reading and identifying statements that were unclear or ambiguous. We addressed these comments below.

*Comment*

**Please find my specific comments delineated in a section-by-section fashion below.**

**Major Comments:**

**Abstract:**

**-Line 26-28: Will development of GBS vaccines be bolstered by this study? Please be more**

**specific as to how this study will contribute to the field of GBS epidemiology.**

*Answer to comment*

In this analysis, we developed statistical models that accounted for the multilevel structure of the data and combined different types of evidence that directly or indirectly inform epidemiologically relevant parameters. These models could be combined with analyses of other steps of the natural history of invasive GBS disease (e.g., case fatality risk and risk of neurodevelopmental impairment in survivors) to generate global estimates of the burden, that will include numbers of deaths and long-term impact (indeed we have a funded programme of work ourselves to produce such estimates). These burden estimates can then be used in the estimation of the cost-effectiveness of future maternal GBS vaccines and other GBS-related interventions, which will be important to guide the prioritization and clinical development.

We have now modified the following sentence in the *Abstract*:

*“Overall, we believe our method will contribute to a more comprehensive* ***quantification*** *of the global burden of this disease,* ***inform cost-effectiveness assessments of potential maternal GBS vaccines*** *and* ***identify*** *key areas where data are necessary.”*

*Comment*

**Background:**

**The authors should make a better case for inferring the incidence of invasive GBS disease based on alternative streams of information. Invasive GBS disease is a life-threatening illness; thus, one would expect that conclusive clinical information would readily available and highly representative of the true incidence of this disease. Please address this (either in the Abstract or further in the Background section).**

*Answer to comment*

Under-reporting and under-ascertainment are pervasive problems in infectious disease burden estimation (for examples in the context of surveillance see Gibbons et al. *Measuring underreporting and under-ascertainment in infectious disease datasets: a comparison of methods*. BMC Public Health 2014). However, as articulated by the reviewer, for a clinical condition as severe as invasive GBS disease one would expect epidemiological information is readily available. Unfortunately, as repeatedly argued in manuscripts presenting direct estimates of incidence of invasive GBS disease (see references below and the main text), under-ascertainment and/or under-reporting might lead to considerable under-estimation of incidence. Some of the key reasons for incidence under-estimation are illustrated below in a figure from the paper by Seale and colleagues (*Estimates of the Burden of Group B Streptococcal Disease Worldwide for Pregnant Women, Stillbirths, and Children* CID 2017)

Firstly, while infant mortality due to sepsis/meningitis can usually be diagnosed through verbal autopsy in community studies in many low- and middle-income settings, there are limited resources to identify the aetiology of such disease cases outside clinical settings or special studies. Furthermore, in a review by Dagnew and colleagues (*Variation in reported neonatal group B streptococcal disease incidence in developing countries*. CID 2012), who summarized results of invasive GBS disease studies performed in low- and middle-income countries, it was argued that poor access to care and rapid disease progression (Benitz and Long. *The Holy Grail of Ascertainment of Early-Onset Neonatal Sepsis*. J Pediatr. 2019) might lead to under-estimation of incidence. In that review, of the 20 GBS incidence studies described, only one study used community-based case finding, while others relied primarily on hospital admission data (see **Table 1** of the manuscript by Dagnew et al). The significance of this observation is made clear by findings of a recent meta-analysis on all-cause neonatal sepsis incidence (Fleischmann et al. *Global incidence and mortality of neonatal sepsis: a systematic review and meta-analysis.* Arch Dis Child 2020), where estimates were considerably higher in community-based versus hospital-based studies. Indeed, these arguments are also supported by findings of the ANISA study (Saha et al. *Causes and incidence of community-acquired serious infections among young children in south Asia (ANISA): an observational cohort study*. Lancet 2018), with prospective neonatal follow-up, where two thirds of deaths in babies not registered in the study occurred in the first 24 hours of life. Additional, indirect evidence for under-ascertainment comes from a meta-analysis that described low care seeking behaviour in these countries for neonatal illnesses (Herbert et al. *Care Seeking for Neonatal Illness in Low- and Middle-Income Countries: A Systematic Review.* PLOS Medicine 2012). Non-optimal diagnostics have also been shown to influence incidence estimates (in the review by Dagnew and colleagues).

Under-estimation has also been described in invasive GBS disease incidence studies performed in high income countries. For example, in an early study that relied on reporting of invasive GBS disease cases, authors estimated under-reporting of 44% and 21% from paediatricians and microbiologists, respectively, and that the number of cases might have been ~20% higher than reported (Heath et al. *Group B streptococcal disease in UK and Irish infants younger than 90 days*. Lancet 2004). In a recent study in the United Kingdom that used a series of capture methods for prospective surveillance, microbiological data from reference laboratories were only available in 53% of the cases (O’Sullivan et al. *Group B streptococcal disease in UK and Irish infants younger than 90 days, 2014–15: a prospective surveillance study.* Lancet Infectious Diseases 2018). In another large prospective study, in Italy, Berardi argued that low sensitivity of systemic cultures, in particular after intrapartum prophylaxis, could have led to under-estimation of incidence, in addition to under-reporting by medical staff (Berardi et al. *Group B streptococcal infections in a northern region of Italy*. Pediatrics 2007). Importantly, variation in *de facto* compliance with intrapartum prophylaxis policy in incidence studies complicates comparisons and extrapolation of results to areas with different prophylaxis policies.

We have now included additional statements in the *Background* section to highlight this issue:

*“It was argued that under- ~~reporting~~* ***estimation*** *~~of early-onset infections~~ is common in incidence studies (5).* ***For example, in an early study in the United Kingdom, authors estimated under-reporting of 44% and 21% from paediatricians and microbiologists, respectively, and that the number of cases might have been ~20% higher than reported (REF: Heath et al, Lancet 2004).*** *Consistent with this* ***concern on case capture****, a recent study with prospective enhanced surveillance that captured data on iGBSd in the first 3 months of life using a variety of approaches found that for 53% of the identified cases microbiological data from reference laboratories were not available (8). As suggested in* ***a*** *~~meta-analysis~~* ***review*** *of iGBSd incidence studies* ***performed in low and middle-income countries*** *(4), under-~~reporting~~* ***estimation*** *could also be related to* ***limited access to health care facilities and*** *the fact that a baby’s clinical condition might deteriorate rapidly, leading to fatal outcome before samples are collected.* ***Indeed, in another review (REF: Madrid et al. CID 2017), the proportion of early-onset iGBS disease cases diagnosed in the first 24 hours of life was higher in high income, versus low income, countries (74% and 31% respectively).****”*

Of note, although the current work attempts to address under-estimation of invasive GBS disease incidence, an important and still neglected aspect of iGBSd epidemiology are probable, unconfirmed cases. For example, in France, while the incidence of culture-confirmed early-onset invasive GBS disease in babies was 0.75 cases per 1,000 births, probable GBS cases were estimated to have incidence that was six times higher (Kuhn et al. *Incidence and distribution of pathogens in early‐onset neonatal sepsis in the era of antenatal antibiotics*. Paediatr Perinat Epidemiol. 2010). Future improvements of our approach should include estimation of numbers of cases of presumed invasive GBS infection; for this, a systematic assessment of data on confirmed versus presumed invasive GBS disease cases would be required.

*Comment*

**Also, the authors should expand on the need for inferred incidence of invasive GBS disease (i.e. what can this information be used for).**

*Answer to comment*

In terms of estimation, in the answer to the previous comment, we provided a series of arguments on the need to infer incidence of invasive GBS disease. From a broader public health perspective, our model on the incidence of invasive GBS disease will inform a larger effort to estimate global acute and long-term impact of GBS disease and will be used in cost-effectiveness analyses of preventative strategies, including maternal vaccines. We have now included the following statement to emphasize the importance of reliable estimates of invasive GBS disease incidence:

*“****Quantification of*** *~~iGBSd~~* ***incidence****, and ~~coherently estimating the~~ associated uncertainty,* ***is the first step to estimate the global burden of iGBSd, including the burden linked to long-term consequences (REF: Kohli-Lynch et al. CID 2017), and is*** *essential for priority setting around investment in preventive interventions such as GBS vaccines (9, 10) and roll out of antibiotic prophylaxis (11). Here, we use Bayesian methods to estimate the number of early-onset iGBSd cases in countries with data on maternal GBS colonization prevalence.”*

*Comment*

**Results:**

**-Figure 5: It is unclear to me whether the data are represented as absolute counts or relative cases/population. If the former, I recommend changing the graph to report cases per population.**

*Answer to comment*

We have now modified the y-axis label to ‘cases per population’ (see updated Figure 5 below) and the figure legend:

*“Estimated* ***absolute*** *numbers of early-onset invasive GBS disease cases (~~EOGBS,~~ y-axis).”*

Also, note that in response to a comment by reviewer #2 we have included a similar figure in the Supplementary Appendix that shows incidence in the y-axis.

*Comment*

**Discussion:**

**-How does this study address issues of population-specific differences within a country? Would this study be able to estimate incidence of invasive GBS disease in regions where specific ethnic and cultural minorities dominate?**

**-Overall, the discussion appropriately contextualizes the findings of this manuscript.**

*Answer to comment*

It is a limitation of our analysis not to describe subnational variation in invasive GBS disease incidence. This is partially due to the fact that data used in this analysis, from a series of literature reviews (see manuscript for references), are not stratified by subnational regions, districts or provinces. Indeed, there was a large number of studies on maternal GBS colonisation, and for some countries, such as China or India, the number of country-specific studies might have been sufficient to reliably inform within-country variation. Although our models were developed to estimate GBS incidence at the national level, the same structure could be adapted to describe variation in the subnational (regional) level. This approach could inform the reviewer’s question on the incidence in regions where specific ethnic and cultural minorities dominate.

*Comment*

**Minor Comments**

**Line 38-39: The parallel structure of this sentence seems incorrect or at least awkward. Please reword this sentence.**

*Answer to comment*

We modified the sentence in response to a previous comment:

*“Overall, we believe our method will contribute to a more comprehensive* ***quantification*** *of the global burden of this disease,* ***inform cost-effectiveness assessments of potential maternal GBS vaccines*** *and* ***identify*** *key areas where data are necessary.”*

*Comment*

**Line 372-375: Parallel structure of this sentence is lacking.**

*Answer to comment*

We have modified the sentence:

*“For the estimation of country-level maternal GBS colonization prevalence, we used a hierarchical model, which allowed partial pooling of data from different countries and* ***also allowed*** *to directly account for between-study differences in GBS detection methods.”*

*Comment*

**Line 412-415: Please shorten this sentence or split it in two.**

*Answer to comment*

We have shortened and split the sentence:

*“Another likely mechanism of under-estimation of early-onset iGBSd incidence~~, that might apply to both incidence studies and studies that assess the risk of disease in babies born to GBS-colonized mothers, in particular those with pregnant women receiving prophylactic antibiotics,~~ relates to the sensitivity of microbiological methods.* ***This might apply to both incidence studies and studies that assess the risk of disease in babies born to GBS-colonized mothers.****”*

**Reviewer #2**

*Comment*

**The authors aimed to estimate the country-level incidence of EOGBS. The have also updated published methods using a hierarchical Bayesian approach. First, the statistical methods seem very appropriate for the task and I commend the authors the thought and effort that has gone into this work. It will represent an important contribution to the field of newborn health.**

**•The use of antibiotic coverage for children with respiratory infections seems to be a poor proxy for antibiotic use among pregnant women (lines 185-186). I understand the limitations in relevant data and the need to account for antibiotic use. However, the authors could discuss the implications, including directionality, associated with using this proxy.**

*Answer to comment*

Inclusion of antibiotic coverage in the analysis was based on the reasoning that recent antibiotic use could have a general effect on bacterial colonisation of maternal genito-urinary tract (Kuperman and Koren. *Antibiotic use during pregnancy: how bad is it?* BMC Medicine 2016). Country-level coverage of antibiotic use for lower respiratory infections in children is used as proxy for antibiotic use during pregnancy; however, as noted by the reviewer, it is possible that this is not a close proxy, and it might be also correlated with other country-level factors that might influence maternal GBS colonisation prevalence. We have now included a statement in the *Discussion* section about this (see below).

Importantly, even if data on a more direct proxy of antibiotic use during pregnancy become available, an association between such a variable and GBS colonisation prevalence at the national level should not be interpreted as evidence for individual level causal effect of antibiotic use on GBS colonisation. Indeed, although for some questions group-level analyses are necessary for epidemiological understanding (see for examples Figures 1 and 2 of the paper by Mervyn Susser. *The Logic in Ecological: I. The Logic of Analysis.* American Journal of Public Health 1994), group-level associations might not reflect individual-level associations or causal effects (see an example in Gelman et al. *Models, assumptions and model checking in ecological regressions.* J. R. Statist. Soc. A 2001; and Greenland*. Ecologic versus individual-level sources of bias in ecologic estimates of contextual health effects*. International Journal of Epidemiology 2001, and Greenland. *A review of multi-level theory for ecologic analyses.* Statistics in Medicine 2002, for discussions on the related issues). We have now modified these sentences in the *Discussion* section to highlight this:

*“Antibiotics use during LRIs, on the other hand, was included in our analysis as a surrogate for antibiotics availability and use in different countries, which could influence frequencies of bacteria carriage, in particular GBS.* ***Our results suggest an association between this variable and maternal GBS colonisation prevalence at the national level, which should not be interpreted as causal at the individual level [REF:*** ***Greenland 2001, and 2002] and could be related to other country-level differences in medical practices if this variable is not a close proxy for antibiotic use during pregnancy.****”*

*Comment*

**•Related to antibiotic use, Figure 2 seems to indicate that median colonization prevalence from the literature is lowest in largely low- and middle-income countries. Am I reading this correctly? I would assume this has to do with exposure to antibiotics before being sampled in such countries. But I would be interested to hear the author’s perspectives on this. Would this need to be accounted for?**

*Answer to comment*

As indicated above, there is a biological basis in the assumption that antibiotic use can influence bacterial colonisation during pregnancy, and it is possible that differences in antibiotic exposure might have contributed to the pattern alluded by the reviewer. However, there are many other differences between low- and middle-income countries and high-income countries that could have contribute to this. Relevant to this question, as mentioned in the legend of Figure 2, countries were ordered in the x-axis by the median of the observed GBS prevalences in the studies; since this figure shows observed data, rather than Bayesian estimates, countries where a higher proportion of studies were performed with sensitive diagnostic combinations might appear in this figure with higher medians of observed GBS prevalence. In our statistical analysis, we included regression coefficients for the diagnostic methods used in each study to account for these between study differences in sensitivity.

*Comment*

**•Last, would it be possible to extend these findings to other countries? Could the authors comment on what might be needed extend this model to cover additional countries?**

*Answer to comment*

One of the objectives in developing the hierarchical model with country-level covariates was to estimate country-level maternal GBS colonisation prevalence in countries without data. For that, we will sample the logit-prevalence in additional countries, $\mu_{j-new}$, from the predictive distribution by using posterior samples of $\mu_{g}$ , $\beta_{c}$ , $\sigma_{c}$ (see the *Results* section for definitions of these parameters), and country-specific covariate values $X_{j-new}^{c}$ .

$\mu_{j-new}\sim Normal (\mu_{g} + \beta_{c} X_{j-new}^{c}, \sigma_{c}^{2} )$

Estimates for countries with no data will be more uncertain than estimates for countries where studies have been performed, as the former incorporate both uncertainty in the parameters listed above as well as between country variation embodied in $\sigma_{c}$ . We have now modified the paragraph in the *Discussion* section related to this:

*“As one of the objectives in developing this model was to, in future analyses, estimate GBS colonization prevalence in countries where no studies have been performed, we also included country-level covariates in our model. The following variables were associated with country-level maternal GBS colonization prevalence: HIV prevalence, antibiotics use during lower respiratory infections (LRIs) in children, gross national income per capita, neonatal mortality and obesity prevalence. At the individual level, HIV infection has been linked to GBS infection phenotypes, although a recent meta-analysis suggests that HIV infection does not have a significant effect on maternal GBS carriage (20). Antibiotics use during LRIs, on the other hand, was included in our analysis as a surrogate for antibiotics availability and use in different countries, which could influence frequencies of bacteria carriage, in particular GBS.* ***Our results suggest an association between this variable and maternal GBS colonisation prevalence at the national level, which should not be interpreted as causal at the individual level [REF:*** ***Greenland 2001] and could be related to other country-level differences in medical practices if this variable is not a close proxy for antibiotic use during pregnancy.*** *Gross national income per capita is likely associated with other factors that directly influence risk of colonization and could represent distal determinants of disease as defined by Victora and colleagues (21). Furthermore, the association between obesity prevalence and country-level maternal GBS prevalence is supported by previous studies that demonstrate an association at the individual level (22).* ***In future analyses, the posterior distributions of the coefficients for these different variables will be used in the estimation of maternal GBS colonisation prevalence for countries without data, which will also incorporate the unexplained between country variation represented by the scale parameter*** $\boldsymbol{\sigma}_{\boldsymbol{c}}$***.”***

*Comment*

**Minor points**

**•On line 57, the authors should provide a citation for Group B Streptococcus being a major cause of morbidity or mortality. Or tone down this language.**

*Answer to comment*

We have modified this sentence:

*“Infection by Group B Streptococcus (GBS), a gram-positive bacterium that colonizes the genital and gastrointestinal tracts (1, 2), ~~is a major cause of~~* ***causes*** *morbidity and mortality in babies in the first months of life.”*

*Comment*

**•On lines 60-61, the authors state that “only few studies had tried to assess…” But only one reference is given. Reference 5 also corresponds with a multi-country analysis and should be provided here.**

*Answer to comment*

We have now added Reference 5 to that sentence.

*Comment*

**•Beginning on line 65, it would help the clarity to note who used knowledge on the pathogenesis. Was this Seale, et al?**

*Answer to comment*

We modified this sentence:

*“In the most comprehensive multi-country analysis to date, Seale and colleagues combined data from different types of studies to estimate the global number of invasive GBS disease (iGBSd) cases in babies (5).* ***In that analysis,*** *knowledge on the pathogenesis of iGBSd was used to identify studies that provide relevant information: ...”*

*Comment*

**•For figure 5, could the authors consider a figure that captures incidence as well? Most countries seem to be right at 0 EOGBS cases, but I wonder how this would change if it were per 1000 live births.**

*Answer to comment*

We have now included in the Supplementary Appendix **Figure S**, which shows incidence of early-onset invasive GBS cases per 1000 live births (see below).
